# Comment on “Failure of the Pipeline Embolization Device in Posterior Communicating Artery Aneurysms Associated with a Fetal Posterior Cerebral Artery”

**DOI:** 10.1155/2017/1685358

**Published:** 2017-03-15

**Authors:** Visish M. Srinivasan, Peter Kan

**Affiliations:** Department of Neurosurgery, Baylor College of Medicine, Houston, TX, USA

We read with interest the article by Zanaty et al. “Failure of the Pipeline Embolization Device in Posterior Communicating Artery Aneurysms Associated with a Fetal Posterior Cerebral Artery” [1].

The authors presented a case series of 4 patients who had been treated with the pipeline embolization device for posterior communicating artery (PCoA) aneurysms across several institutions. However, their review of the previously published literature is incomplete and indeed this phenomenon has been described by our own series of 4 patients several months earlier [2]. Soon, thereafter, it was described by Tsang et al. [3], and followed by the recent article by Zanaty et al. These publications were likely in development simultaneously, but we wish to point out the previously published literature on the topic and reinforce the phenomenon observed across the 3 series.

All 12 cases (4 in each series) involved posterior communicating artery aneurysms that incorporated a fetal origin of the posterior cerebral artery (PCA). The large flow demand of the fetal PCA causes flow across the aneurysm and into the distal PCA territory, preventing successful flow diversion. Without diversion of flow, the aneurysm remains patent and treatment fails. The same concept applies to other aneurysms incorporating “end vessels”; we recently presented this concept at the AANS 2016 meeting [4] and subsequently published it in* Journal of Neurosurgery* [5].
